# Supplementary material for: A bromodomain–DNA interaction facilitates acetylation-dependent bivalent nucleosome recognition by the BET protein BRDT
Source: Nat Commun. 2016 Dec 19;7:13855. doi: 10.1038/ncomms13855 (PMC5187433; doi:10.1038/ncomms13855)
Supplement: Supplementary Information — Supplementary Figure 1-12, Supplementary Table 1 and Supplementary References. [file ncomms13855-s1.pdf]

Supplementary Figure 1

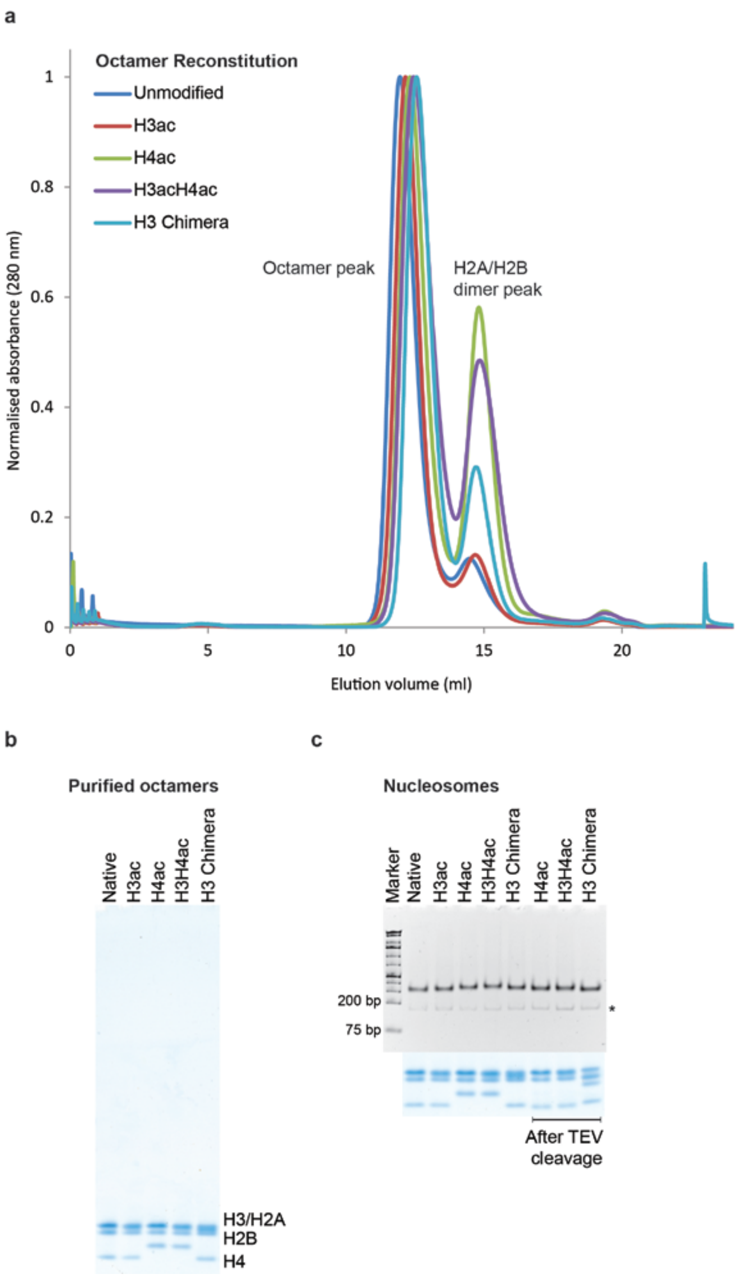

Supplementary Figure 1. *In vitro* reconstitution of acetylated nucleosomes. (**a**) Size exclusion (HiLoad 16/600 Superdex 200 pg) gel filtration profiles showing the purification of unmodified and acetylated histone octamers (as indicated), from excess H2A/H2B dimers. (**b**) SDS-PAGE analysis of purified octamers (NuPAGE™ Novex™ 4-12% Bis-Tris protein gel). (**c**) Native-PAGE (4% TAE gel) and SDS-PAGE (as for (**b**)) analysis of purified mononucleosomes. Nucleosomes containing the acetylated H4 tail (H4K5_{ac}K8_{ac}) are shown before and after TEV cleavage performed for 2 hours at 30°C. *Small excess of unincorporated 167 bp Widom DNA.

Supplementary Figure 2

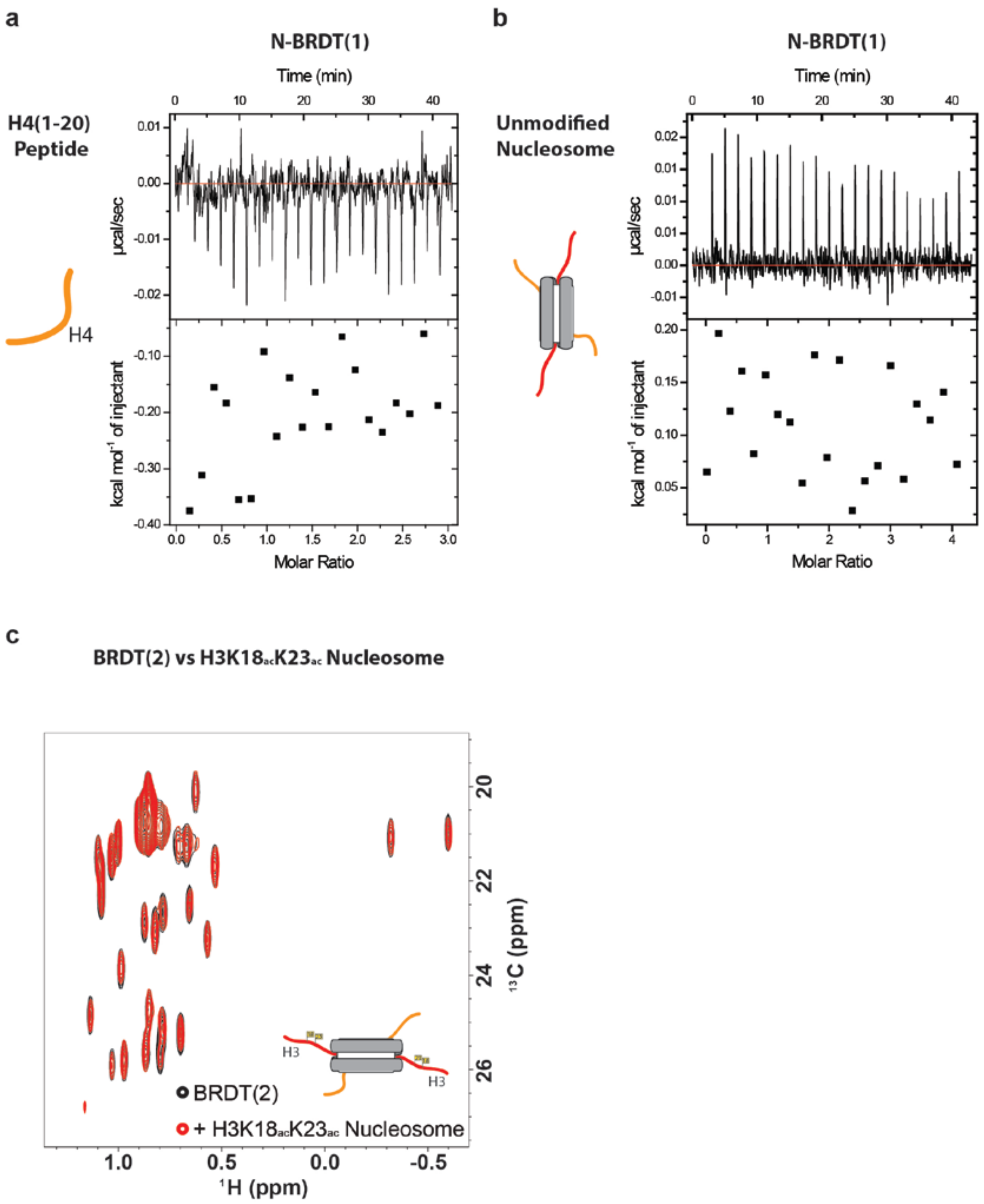

Supplementary Figure 2. BRDT-BD1 requires acetylation for binding to histone peptides or nucleosomes, whilst BRDT-BD2 cannot interact with acetylated nucleosomes. ITC profiles for N-BRDT(1) interactions with (**a**) unmodified histone H4 tail peptides or (**b**) unmodified nucleosomes. (**c**) Overlaid ^{13}C-^{1}H methyl-TROSY spectra of BRDT(2) alone and BRDT(2) in the presence of a 2-fold molar excess of $H3K18_{ac}K23_{ac}$ nucleosomes. Spectra colored as indicated.

Supplementary Figure 3

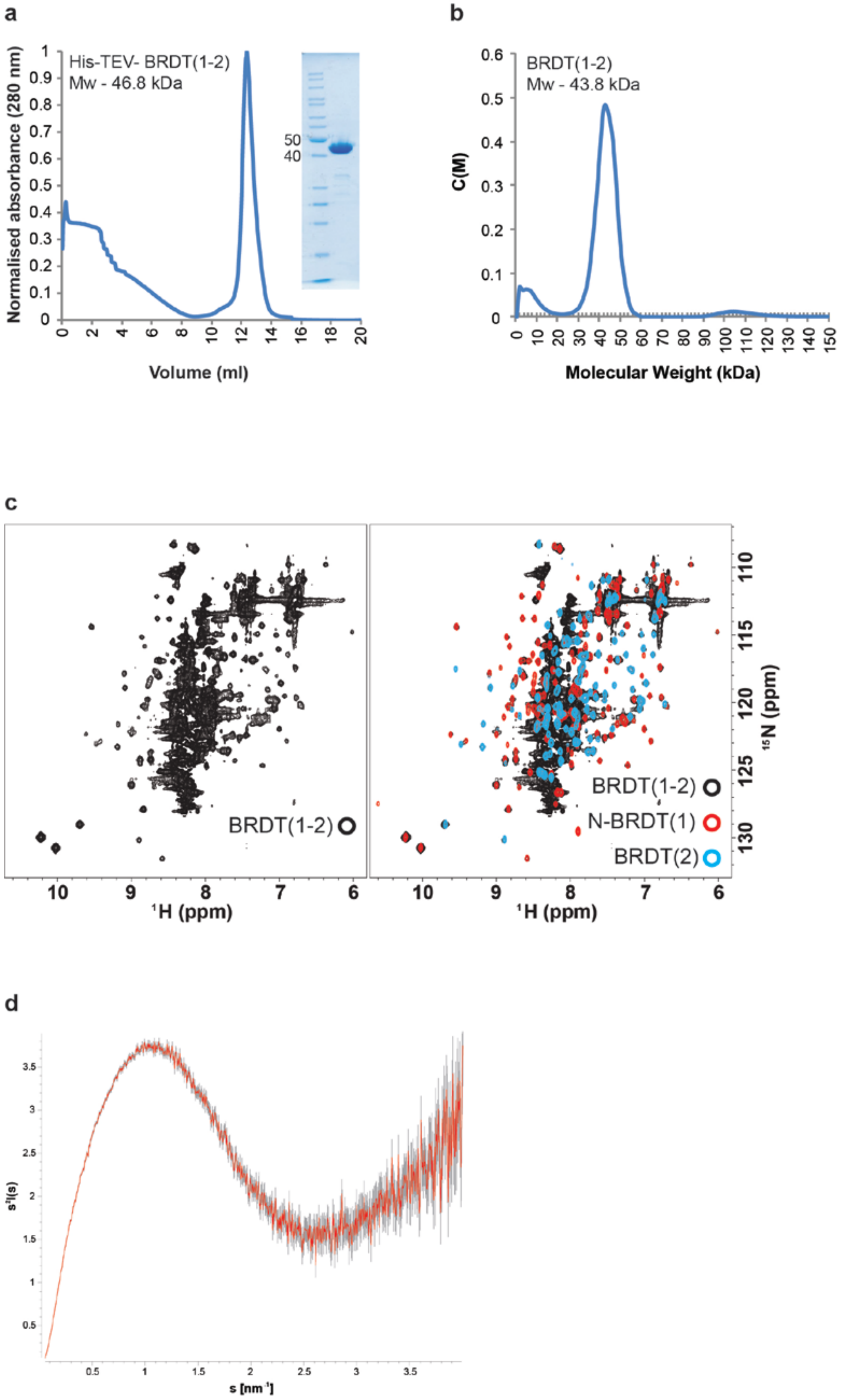

Supplementary Figure 3. BRDT(1-2) is a stable, monomeric, elongated molecule with two folded bromodomains at either end of a disordered linker. (**a**) Chromatogram from size exclusion purification of His-TEV-BRDT(1-2) on a Superdex 200 HR 10/30 gel filtration column. Coomassie stained SDS-PAGE gel shows the final purity of the sample used for SAXS. (**b**) Analytical ultracentrifugation (AUC) analysis of BRDT(1-2). The predicted Mw of BRDT(1-2) is 43.8 kDa, as shown. The calculated weight average of the integrated AUC peak is 43.5 kDa. (**c**) ^{1}H, ^{15}N HSQC spectrum of BRDT(1-2) shown beside the same spectrum overlaid with the equivalent spectra of N-BRDT(1) and BRDT(2), colored as indicated. (**d**) SAXS analysis of BRDT(1-2). The radius of gyration (R_g) = 49 +/- 9 Å and D_{max} of > 160 Å derived from the SAXS data of BRDT(1-2) indicate a large average distance between the two bromodomains. In structures with random linker conformations and a corresponding R_g, the distance between the center of mass of the two bromodomains is > 80 Å. The Kratky plot shows a clear bell-shaped curve indicative of structured proteins; however, the curve increases asymptotically as expected for a non-structured protein. Thus the data confirm the existence of an unstructured linker connecting the two bromodomains.

Supplementary Figure 4

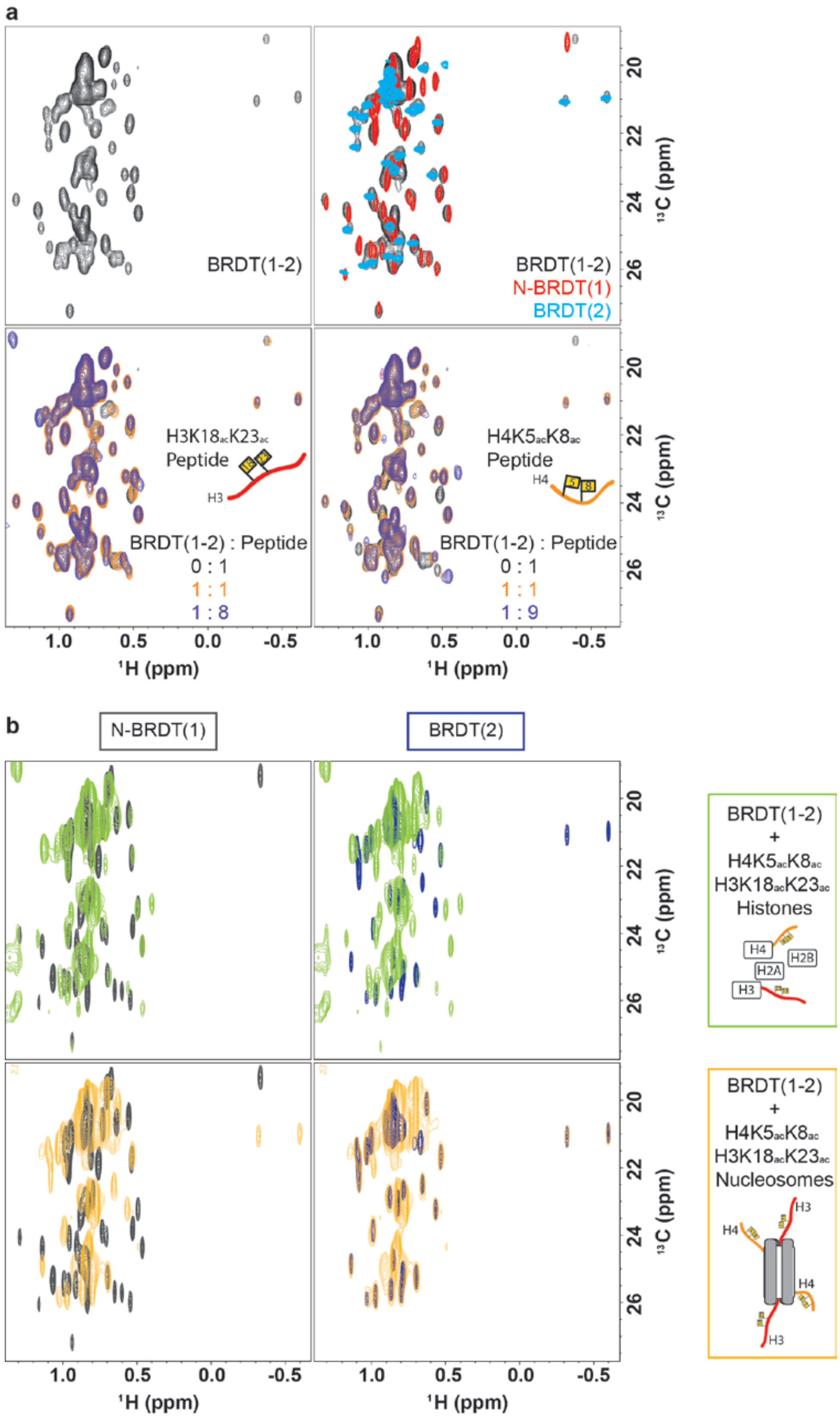

Supplementary Figure 4. The BRDT bromodomains have low specificity for $H4K5_{ac}K8_{ac}$ and $H3K18_{ac}K23_{ac}$ peptides. (**a**) Top panels: ^{13}C-^{1}H methyl-TROSY spectrum of leucine and valine labeled BRDT(1-2), shown beside the same spectrum overlaid with the equivalent N-BRDT(1) and BRDT(2) spectra. Bottom panels: Overlaid ^{13}C-^{1}H methyl-TROSY spectra of BRDT(1-2) in the presence of increasing concentrations of $H3K18_{ac}K23_{ac}$ or $H4K5_{ac}K8_{ac}$ peptides, as indicated. (**b**) ^{13}C-^{1}H methyl-TROSY spectra of leucine and valine labeled N-BRDT(1)(black) or BRDT(2)(blue) overlaid with equivalent spectra of BRDT(1-2) in the presence of $H4K5_{ac}K8_{ac}$/$H3K18_{ac}K23_{ac}$ double modified histones (green) or nucleosomes (yellow).

Supplementary Figure 5

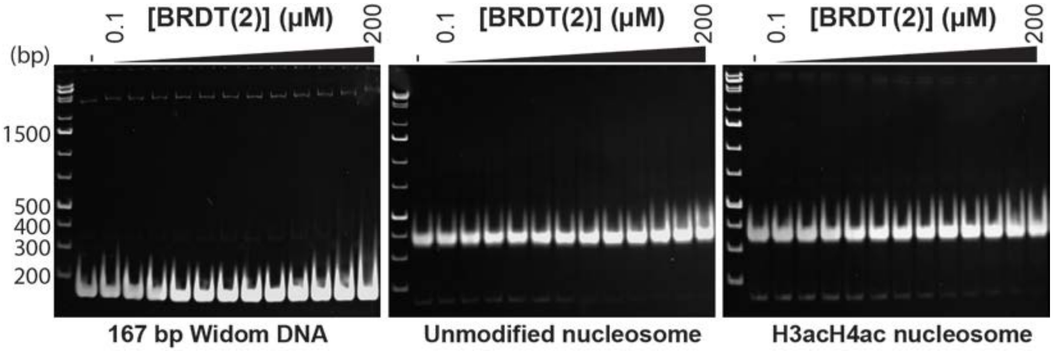

Supplementary Figure 5. BRDT cannot interact with DNA or nucleosomes through BD2. 167 bp Widom DNA, unmodified nucleosomes or $H4K5_{ac}K8_{ac}/H3K18_{ac}K23_{ac}$ double modified nucleosomes (0.5 μM) were mixed with increasing concentrations of BRDT(2) constructs in a final volume of 4 μl and incubated for 30 minutes prior to native-PAGE electrophoresis (4^oC) and visualization with ethidium bromide staining.

Supplementary Figure 6

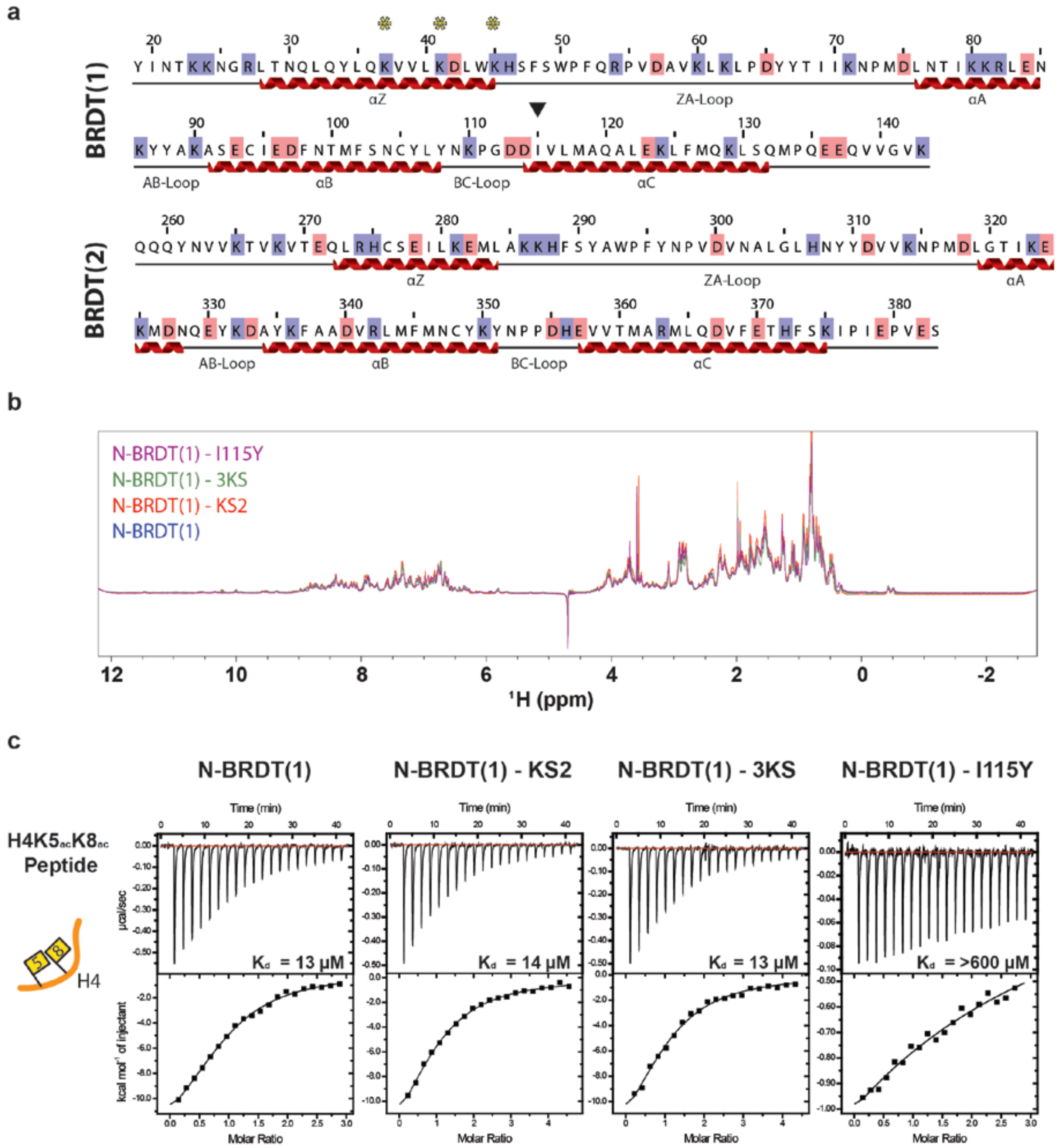

Supplementary Figure 6. Mutation of N-BRDT(1) DNA binding lysine residues does not interfere with bromodomain folding or histone peptide binding. (**a**) Sequences and structural features of BRDT-BD1 and BRDT-BD2 with highlighted acidic (red) and basic (blue) amino acids. Lysines 37, 41 and 45, which were mutated in this study, are highlighted with yellow asterisks. (**b**) Overlaid 1D NMR spectra of N-BRDT(1) mutants. (**c**) ITC profiles for WT and mutant N-BRDT(1) interactions with histone $H4K5_{ac}K8_{ac}$ peptides, as indicated.

Supplementary Figure 7

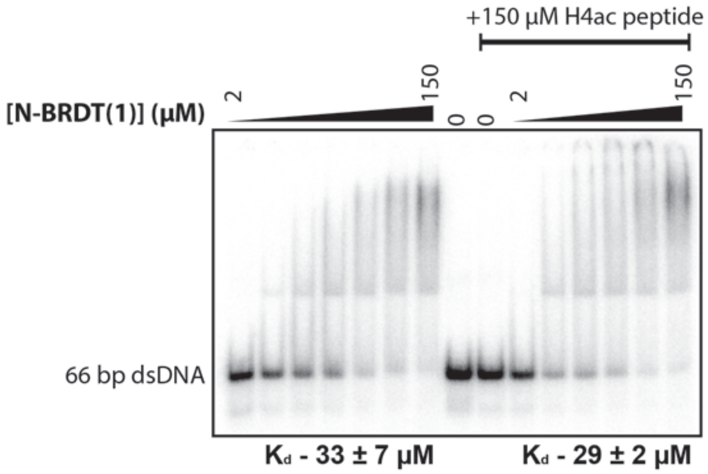

Supplementary Figure 7. The DNA and histone tail binding interfaces of BRDT-BD1 are distinct and independent. EMSA titrations of N-BRDT(1) with radio-labelled 66 bp double stranded DNA. N-BRDT(1) was mixed with either buffer or H4K5$_{ac}$K8$_{ac}$ peptide and incubated for 30 minutes at 4°C. DNA was added giving a final volume of 6 µl and final concentrations of N-BRDT(1) and peptide as shown. Samples were incubated for a further 30 minutes prior to native-PAGE electrophoresis at 4°C. Unbound DNA was quantified using a Typhoon imager with ImageQuant software and the obtained data was used to calculate the indicated affinities.

Supplementary Figure 8

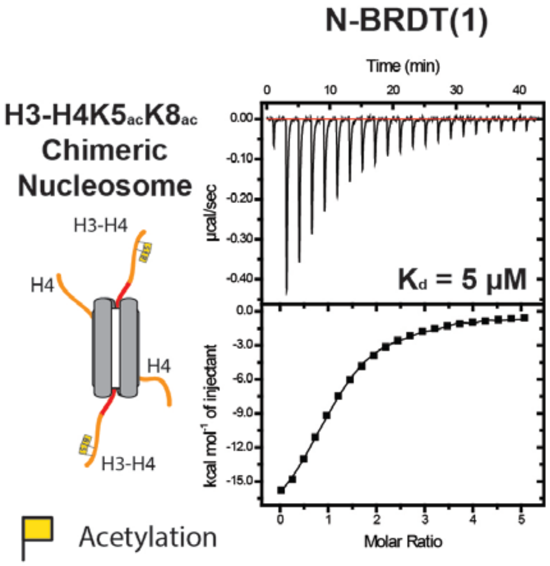

Supplementary Figure 8. The nucleosome structure influences bromodomain specificity for acetylated histone tails. ITC profile for N-BRDT(1) interaction with chimeric histone H3-$H4K5_{ac}K8_{ac}$ nucleosomes.

Supplementary Figure 9

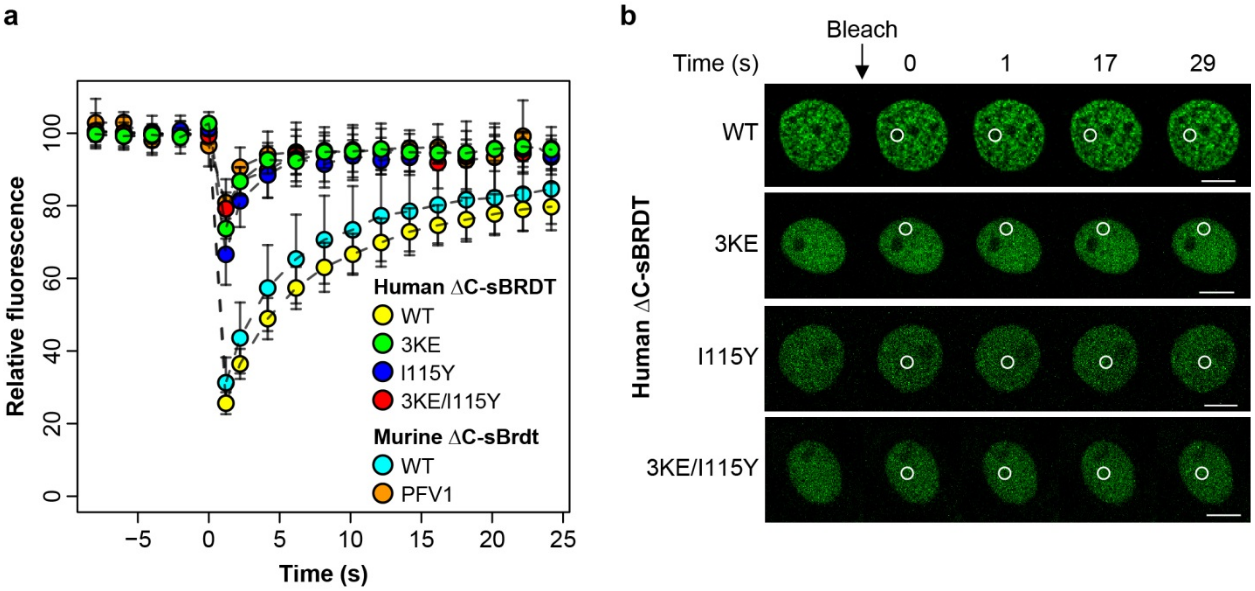

Supplementary Figure 9. FRAP analysis of human and murine ΔC-sBRDT constructs in the presence of TSA-induced histone hyperacetylation (corresponding to **Fig. 6c**). Cos7 cells were transfected by vectors expressing GFP-tagged WT and mutant ΔC-sBRDT constructs and cells were treated with the histone deacetylase inhibitor TSA (100 ng ml^{-1}) to induce histone hyperacetylation. (A) Normalised FRAP curves for each ΔC-sBRDT construct used in this study. Data points represent the mean values of ten individual cells, with the exception of murine WT ΔC-Brdt (n = 9). Error bars show the standard deviation. (B) Representative time-lapse fluorescence microscopy images from FRAP experiments using wild-type and mutant human BRDT constructs. Bleached spots are indicated by white circles. Scale bars are 10 µm.

Supplementary Figure 10

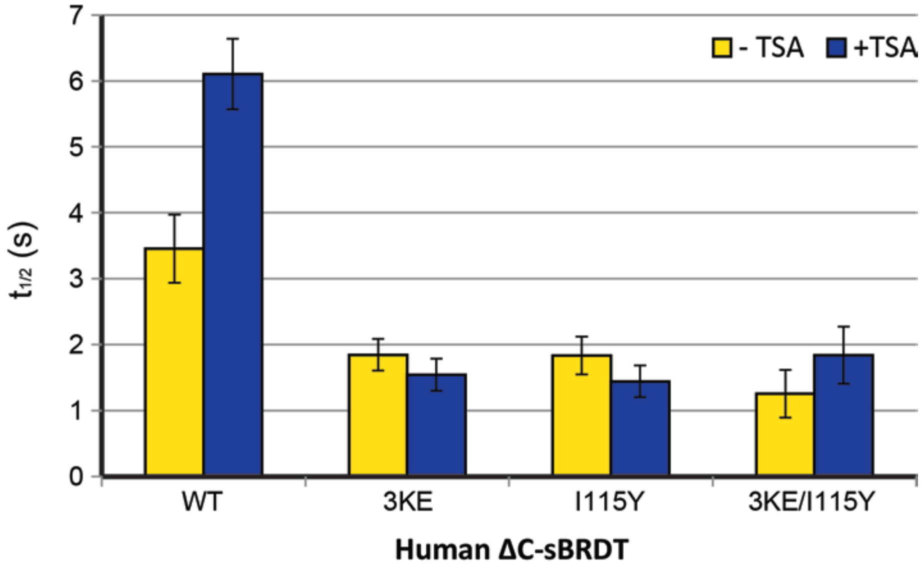

Supplementary Figure 10. BRDT-BD1 DNA binding is important for BRDT's chromatin association and acetylated histone recognition. FRAP analysis of WT and mutant human ΔC-sBRDT constructs in the presence or absence of TSA-induced histone hyperacetylation (100 ng ml^{-1} TSA), as indicated. The fluorescence recovery half-lives are mean values obtained from ten independent cells. Error bars show the standard error of the mean (s.e.m.).

Supplementary Figure 11

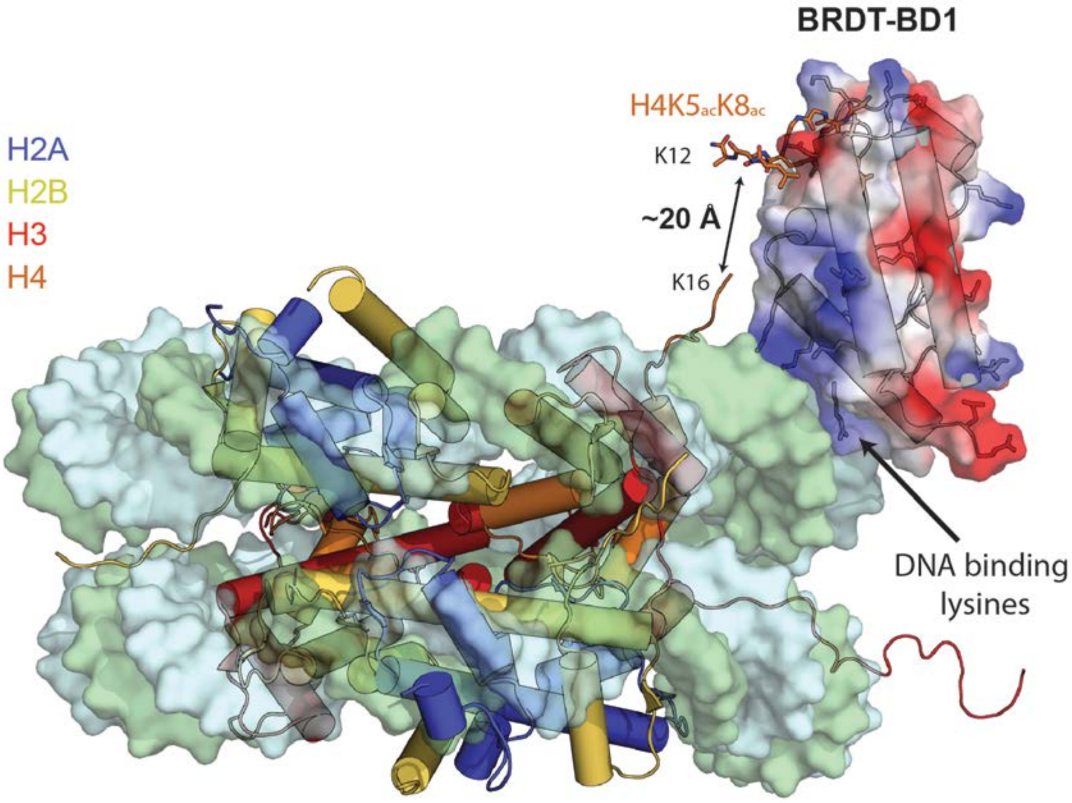

Supplementary Figure 11. The polarized electrostatic surface of BRDT-BD1 aligns BD1 on the nucleosome for acetylated histone H4 tail recognition. Manually aligned model of the crystal structure of murine Brdt-BD1, with bound $H4K5_{ac}K8_{ac}$ peptide (PDB code – 2WP2[1]), aligned to the surface of the mononucleosome (PDB code – 1AOI[2]). BRDT-BD1 has been positioned so that the positive interface containing lysines 37, 41 and 45 are directed towards the DNA. The distance between histone H4 leaving the structure of the nucleosome and the $H4K5_{ac}K8_{ac}$ peptide in the structure of BRDT-BD1 is ~20 Å, with the peptide correctly oriented for interaction.

Supplementary Figure 12

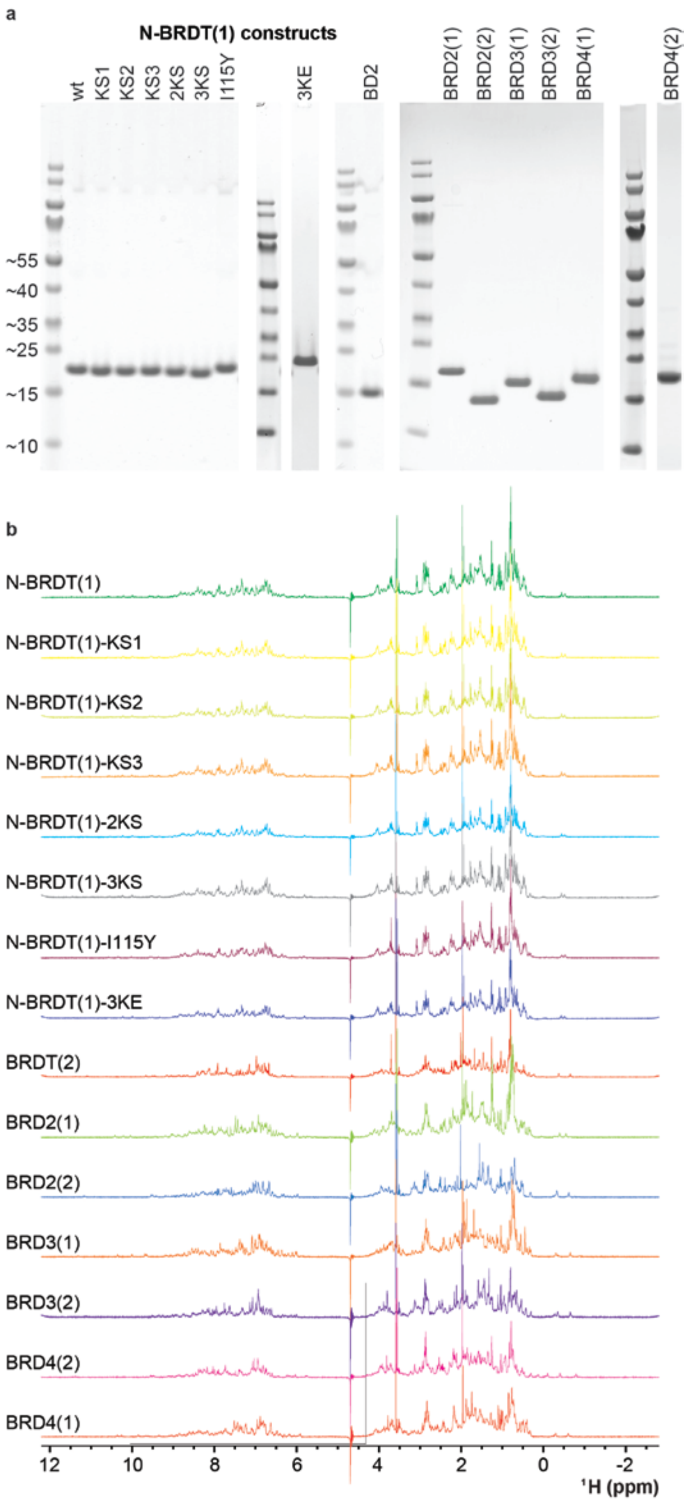

Supplementary Figure 12. Purity and structural integrity of BET bromodomains used in this study. (a) SDS-PAGE analysis and (b) NMR ^{1}H spectra of purified BET bromodomains.

Supplementary Table 1. Summary of ITC data

Fig.	Protein	Peptide/ Nucleosome	Kd (μM)	N	ΔH (kcal/mol)	ΔS (cal/mol/deg)
1b	N-Brdt(1)	H4K5acK8ac Nucleosome	2.0 ± 0.2	2.11 ± 0.04	-20.57	-44.10
1b	N-Brdt(1)	H4K5acK8ac Peptide	12.7 ± 1.0	0.92 ± 0.03	-15.34	-29.90
1c	Brdt(2)	H3K18ac23ac Nucleosome	-	-	-	-
1c	Brdt(2)	H3K18ac23ac Peptide	122.5 ± 6.7	1 ± 0	-3.59	5.66
4d	N-Brdt(1)-3KS	H4K5acK8ac Nucleosome	8.6 ± 0.8	1.98 ± 0.09	-19.57	-43.60
4d	N-Brdt(1)-KS2	H4K5acK8ac Nucleosome	6.8 ± 0.6	1.91 ± 0.06	-13.03	-20.80
5c	Brd4-BD1	H4K5acK8ac Nucleosome	11.8 ± 1.8	1.52 ± 0.13	-17.40	-36.80
5c	Brd4-BD1	H4K5acK8ac Peptide	12.9 ± 2.6	0.98 ± 0.14	-14.02	-25.40
6b	N-Brdt(1) 3KE	H4K5acK8ac Nucleosome	9.5 ± 1.4	2.03 ± 0.12	-13.90	-24.40
6b	N-Brdt(1) 3KE	H4K5acK8ac Peptide	14.2 ± 1.4	0.98 ± 0.07	-19.80	-45.40
S2a	N-Brdt(1)	H4 peptide	-	-	-	-
S2b	N-Brdt(1)	Unmodified Nucleosome	-	-	-	-
S6b	N-Brdt(1) I115Y	H4K5acK8ac Peptide	602.4 ± 55.2	1 ± 0	-12.82	-29.00
S6b	N-Brdt(1)-3KS	H4K5acK8ac Peptide	13.4 ± 1.7	1.02 ± 0.07	-16.92	-35.40
S6b	N-Brdt(1)-KS2	H4K5acK8ac Peptide	14.2 ± 1.1	0.96 ± 0.05	-18.32	-40.30
S8	N-Brdt(1)	Chimeric nucleosome	4.8 ± 0.2	2.11 ± 0.03	-23.17	-54.70

References

1. Moriniere, J. et al. Cooperative binding of two acetylation marks on a histone tail by a single bromodomain. *Nature* **461**, 664-8 (2009).
2. Luger, K., Mader, A.W., Richmond, R.K., Sargent, D.F. & Richmond, T.J. Crystal structure of the nucleosome core particle at 2.8 A resolution. *Nature* **389**, 251-60 (1997).
